# Assessing the staining potential of common sauces on tooth enamel surface

**DOI:** 10.1007/s10266-025-01125-1

**Published:** 2025-05-23

**Authors:** Soyeon Kim, Sri Larnani, Wanki Lee, Napas Lappanakokiat, Van Mai Truong, Wonjoon Moon, Young-Seok Park

**Affiliations:** 1 Department of Oral Anatomy and Dental Research Institute, School of Dentistry, Seoul National University, 101 Daehak-ro, Jongno-gu, Seoul, 03080 Republic of Korea; 2 Center for Future Dentistry, School of Dentistry, Seoul National University, Seoul, Republic of Korea; 3 Faculty of Odonto-Stomatology, University of Health Sciences, Vietnam National University, Ho Chi Minh City, Vietnam; 4 Department of Dental Biomaterials Science and Dental Research Institute, School of Dentistry, Seoul National University, Seoul, Republic of Korea; 5 Department of Integrated Dentistry, School of Dentistry, Seoul National University, Seoul, Republic of Korea

**Keywords:** Tooth discoloration, Dietary chromogens, Enamel staining, Spectrophotometry

## Abstract

Tooth discoloration is a common esthetic concern often linked to dietary exposure to highly pigmented foods and beverages. Although the staining effects of beverages like coffee, tea, and wine have been extensively studied, less attention has been given to the staining potential of everyday condiments. This study aimed to evaluate the staining potential of eight sauces—fish sauce, curry, ketchup, sriracha, and four soy sauce varieties (Jin, Yangjo, Tsuyu, and Sashimi)—on bovine enamel specimens. Color measurements were performed at multiple intervals over 7 days using a spectrophotometer to quantify changes in lightness (L*), redness (a*), and yellowness (b*). The Yangjo soy sauce exhibited the highest staining potential, followed by Sashimi and Jin soy sauces, with all three sauces surpassing the perceptibility and acceptability thresholds within 3 h of staining. Curry showed resistance to staining despite its vivid color and high absorbance, while ketchup and sriracha exhibited minimal discoloration, challenging assumptions about red-pigmented substances. Factors such as pigment composition, acidity, viscosity, and exposure duration can interact in a complex manner to influence discoloration.

## Introduction

Tooth discoloration is a prevalent concern among individuals across various ages and lifestyles. A growing interest in addressing tooth discoloration highlights the need to understand its causes. The discoloration can be broadly categorized into two types with distinct underlying factors: intrinsic and extrinsic. Intrinsic discoloration occurs internally within the dentin or enamel and may result from factors such as aging, the use of specific medications (e.g., tetracycline), traumatic injuries, or genetic conditions such as amelogenesis or dentinogenesis imperfecta [1–3]. In contrast, extrinsic discoloration arises from external factors, primarily the deposition of pigments or chromogens on the tooth surface. These chromogens are abundant in pigmented foods and beverages, such as wine, coffee, and tea, which have been widely studied for their staining effects on both natural teeth and restorative dental materials [4, 5].

However, despite the extensive research on beverages, there is a notable lack of studies examining the staining effects of commonly consumed condiments and sauces, such as soy sauce, ketchup, sriracha, and curry. These sauces are regularly incorporated into diverse diets and contain highly pigmented compounds that could contribute to extrinsic staining. For instance, soy sauce is rich in melanoidin, which is recognized for causing the dark discoloration associated with coffee [6, 7]. Fish sauce also contains melanoidin, although in lower concentrations [8]. Ketchup and sriracha derive their red color from pigments such as lycopene and capsanthin, while curry contains curcumin, a bioactive compound in turmeric known for its strong staining properties [9–11]. Thus, given the frequent consumption of these sauces, consumers and dental professionals need to understand how they impact enamel staining.

The present study aimed to investigate the staining effects of these common sauces on tooth specimens. The samples were immersed in different condiments, and the changes in color were measured using a spectrophotometer. The staining effects were quantified over time using the L*, a*, and b* values, representing the lightness, red–green, and blue–yellow color dimensions, respectively, to track the discoloration. This study provides a direct comparison of the staining potentials of commonly used condiments and information about their influence on tooth appearance over time. The null hypothesis of this study was that there would be no significant differences in staining effects among the tested sauces. The findings of this study can offer insights that may inform consumer choices, dental care practices, and future research on stain prevention and removal strategies.

## Materials and methods

Bovine teeth, comprising central and lateral incisors extracted from the mandibular arch, were sourced from the Korean Traditional Market in Seoul, Korea. The staining potential of various condiments on the teeth was examined using established protocols [12, 13]. The teeth were processed using a bench drilling machine (YDM-13 mm, Yongsoo Precision, Daegu, Korea) equipped with a cylindrical diamond core bit (⌀10 × ⌀8 mm) while maintaining water flow to create enamel disks (8 mm in diameter).

**Fig. 1:**
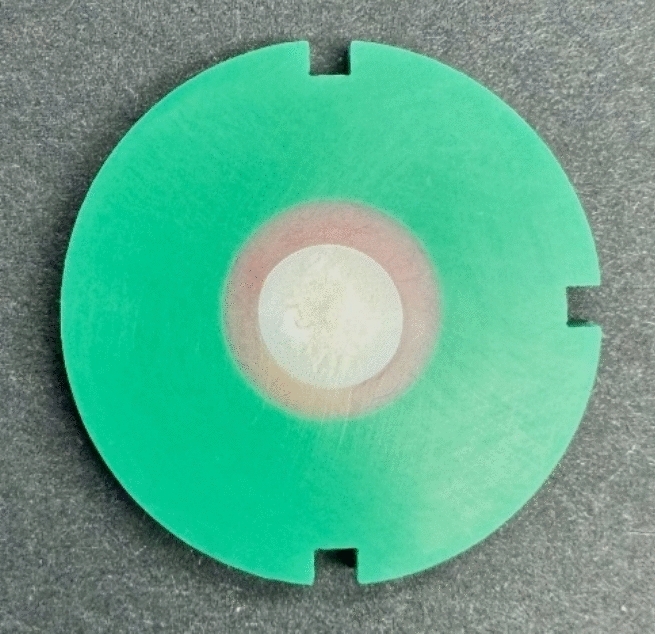
Bovine enamel specimen

The drilled specimens were affixed to custom-designed acrylic rings (⌀30 × ⌀12 × 4 mm) using a self-curing resin (ASCP3000500; Vertex-Dental, Soesterberg, Netherlands) to ensure stability during handling and analysis (Fig. 1). Subsequently, the enamel surfaces were polished with a grinding and polishing machine (LaboPol-5, Struers, Copenhagen, Denmark) using silicon carbide papers in sequential grits (#220, #600, and #1200; R&B, Daejeon, Korea) to achieve uniformity. Specimens meeting the criteria for a Vickers Hardness Number of > 250 and a lightness value (L*) of 75 ± 1 were deemed satisfactory for further testing [6, 12, 14].

Ninety enamel specimens were equally divided into nine groups based on the type of condiment they were immersed in (Table 1): YS (Yangjo soy sauce), SS (Sashimi soy sauce), TS (Tsuyu sauce), JS (Jin soy sauce, F (fish sauce), C (curry), K (ketchup), and S (sriracha). The control (L) group was immersed in distilled water. The sample size was calculated based on an effect size of 0.25, an alpha level of 0.05, and a power of 0.95 (G*Power Version 3.1.9.6). Baseline color measurements were taken before immersion using a desktop spectrophotometer (Ci7600, X-rite Pantone, Grand Rapids, MI, USA). All specimens were immersed directly in the condiments, except for Tsuyu, a concentrated extract. According to the manufacturer’s instructions, Tsuyu required dilution at a ratio of 1:4; thus, it was diluted with distilled water before use. All other sauces were used at their original concentrations.

**Table 1:** Types of sauces and their manufacturers used in the immersion experiment

Sauces	Ingredients	Manufacturer
Jin Soy sauce (JS)	Water, defatted soybeans, high fructose corn syrup, salt, alcohol, wheat, less than 1/10 of 1% sodium benzoate to preserve freshness, yeast extract, sucralose	Sempio, Seoul, Korea
Yangjo Soy sauce (YS)	Water, defatted soybeans, wheat, salt, alcohol, oligosaccharide, licorice extract, yeast extract	Sempio, Seoul, Korea
Sashimi Soy sauce (SS)	Defatted processed soybeans (soybeans (imported)), wheat, salt, sugars (sugar, glucose fructose liquid sugar), alcohol, caramel coloring, seasonings (amino acids, etc.), thickeners (xanthan), sweeteners (licorice, stevia)	Nibishi Shoyu Co., Koga City, Japan
Tsuyu Soy sauce (TS)	Fish extract (35%) (water, dried bonito (fish), dried sardines (fish), water, soy sauce (19%), water, soy beans, wheat, salt), sugar, salt, flavor enhancer e621, alcohol, sea algae extract (water, dried seaweed, salt, alcohol), flavor enhancer: e631, e627	Kikkoman Co., Noda, Japan
Fish sauce (F)	Water, anchovy (Fish) extract 65%, salt, sugar	Tang Sang Hah Co., Chon Buri, Thailand
Curry (C)	Wheat flour, vegetable oils (palm oil, hydrogenated rapeseed oil), salt, Sugar, curry powder, monosodium glutamate, caramel color, malic acid, pepper, chili pepper, garlic, disodium guanylate, disodium inosinate, celery seed, mustard	S&B, Tokyo, Japan
Ketchup (K)	Tomato concentrate from red ripe tomatoes, distilled vinegar, high fructose corn syrup, corn syrup, salt, spice, onion powder, natural flavoring	Kraft Heinz, IL, USA
Sriracha (S)	Chili, sugar, salt, garlic, distilled vinegar, potassium sorbate and sodium bisulfite as preservatives, xanthan gum	Huy Fong Foods, Inc., CA, USA

Absorbance was measured at a wavelength of 416 nm using a Hidex Chameleon spectrophotometer (Hidex Oy, Turku, Finland). Viscosity was assessed using a viscometer (RV-2 T/DV-1/NDJ-8S, W&J Instrument Co., Mudu Town, China) with solutions maintained at room temperature and a rotation speed of 60 rpm. The pH of each solution was measured at room temperature using a pH meter (F-71, HORIBA, Kyoto, Japan).

Color measurements were recorded at 3, 9, 24, and 48 h and 7 days after immersion. The specimens were placed in separate containers with their respective solutions and stored in a light-free chamber at room temperature. Between measurements, the specimens were rinsed with distilled water and kept moist by covering them with a wet paper towel. Before the color measurement, each specimen was dried with a dry paper towel, and measurements were taken promptly to minimize dehydration and potential color changes caused by delays.

The CIEDE2000 color difference formula was used to quantify color differences:

$$\Delta {\text{E}}_{00} \, = \sqrt {\left( {\frac{{\Delta {\text{L}}}}{{{\text{k}}_{{\text{L}}} {\text{S}}_{{\text{L}}} }}} \right)^{2} \, + \left( {\frac{{\Delta {\text{C}}}}{{{\text{k}}_{{\text{C}}} {\text{S}}_{{\text{C}}} }}} \right)^{2} \, + \left( {\frac{{\Delta {\text{H}}}}{{{\text{k}}_{{\text{H}}} {\text{S}}_{{\text{H}}} }}} \right)^{2} \, + {\text{R}}_{{\text{T}}} \left( {\frac{{\Delta {\text{C}}}}{{{\text{k}}_{{\text{C}}} {\text{S}}_{{\text{C}}} }}} \right)\left( {\frac{{\Delta {\text{H}}}}{{{\text{k}}_{{\text{H}}} {\text{S}}_{{\text{H}}} }}} \right)}$$

The formula accounts for the lightness difference (ΔL′) that measures the difference in perceived brightness and is adjusted using the lightness weighting function (S_L_). The chroma difference (ΔC′) represents the difference in color intensity and is adjusted using the chroma weighting function (S_C_) to ensure perceptual uniformity across different chroma levels. The hue difference (ΔH′) quantifies the variation in the hue angle while incorporating a hue weighting function (S_H_) to correct for perceptual distortions. The rotation term (R_T_) corrects the hue perception irregularities in the blue region. Lastly, the scaling factors (k_L_, k_C_, k_H_) were set to 1, as standard viewing conditions were applied.

A two-way analysis of variance (ANOVA) followed by Tukey’s post hoc test was performed on ΔE_00_ values across five time points to evaluate the extent of discoloration among specimens immersed in different sauces and to assess the effects of time, sauce type, and their interaction.

## Results

The null hypothesis was rejected as significant differences in discoloration potential (ΔE_00_) were observed between groups (*p* < 0.001), across time points (*p* < 0.001), and in their interaction (*p* < 0.001). These results indicate that the extent of discoloration varied not only between solutions and over time, but also that the pattern of change differed by solution.

**Fig. 2:**
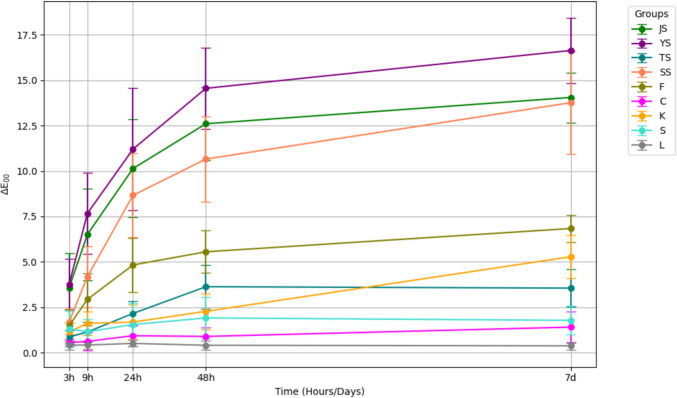
Change in color difference after consecutive immersion in nine different solutions

At the 7-day time point, Yangjo soy sauce exhibited the highest degree of discoloration, significantly greater than all other staining solutions (*p* < 0.001) (Table 2). Sashimi and Jin soy sauces followed in staining intensity, while Curry and Sriracha showed the lowest discoloration values and were not significantly different from one another (*p* = 0.999) (Fig. 2). Other notable comparisons included significantly higher ΔE₀₀ values in Ketchup compared to Curry (*p* < 0.001), and in Tsuyu compared to Sriracha (*p* < 0.001), reflecting varied staining potentials across immersion media.

**Table 2:** Significant pairwise group differences in ΔE₀₀ at 7 days of discoloration

Group	Significant differences
Control	Fish sauce (*p* < 0.0001); Jin (*p* < 0.0001); Ketchup (*p* < 0.0001); Sashimi (*p* < 0.0001); Tsuyu (*p* < 0.0001); Yangjo (*p* < 0.0001)
Curry	Fish sauce (*p* < 0.0001); Jin (*p* < 0.0001); Ketchup (*p* < 0.0001); Sashimi (*p* < 0.0001); Tsuyu (*p* = 0.0083); Yangjo (*p* < 0.0001)
Fishsauce	Jin (*p* < 0.0001); Sashimi (*p* < 0.0001); Sriracha (*p* < 0.0001); Tsuyu (*p* < 0.0001); Yangjo (*p* < 0.0001)
Jin	Ketchup (*p* < 0.0001); Sriracha (*p* < 0.0001); Tsuyu (*p* < 0.0001); Yangjo (*p* = 0.0005)
Ketchup	Sashimi (*p* < 0.0001); Sriracha (*p* < 0.0001); Yangjo (*p* < 0.0001)
Sashimi	Sriracha (*p* < 0.0001); Tsuyu (*p* < 0.0001); Yangjo (*p* = 0.0001)
Sriracha	Yangjo (*p* < 0.0001)
Tsuyu	Yangjo (*p* < 0.0001)

**Fig. 3:**
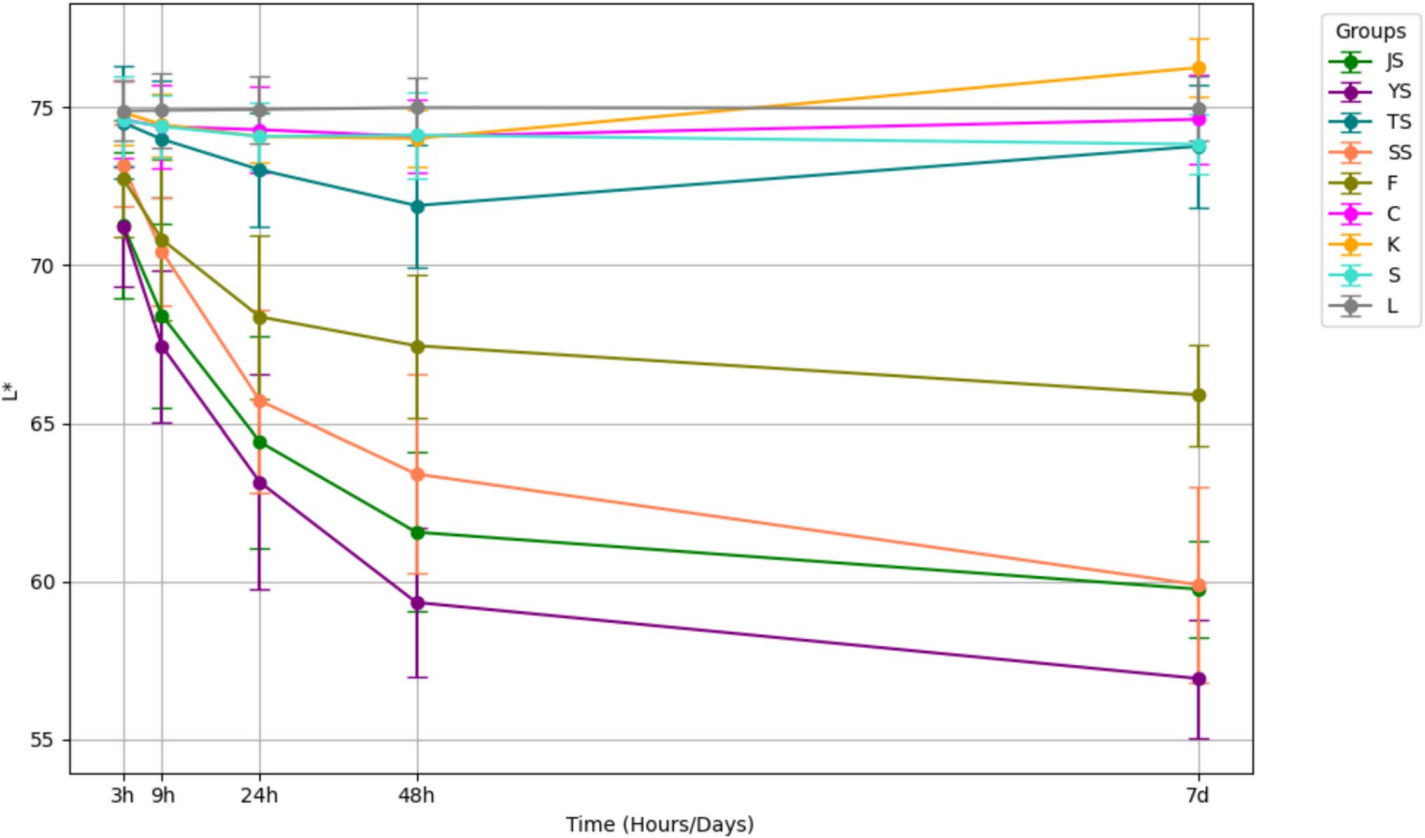
Change in L* values after consecutive immersion in nine different solutions

**Fig. 4:**
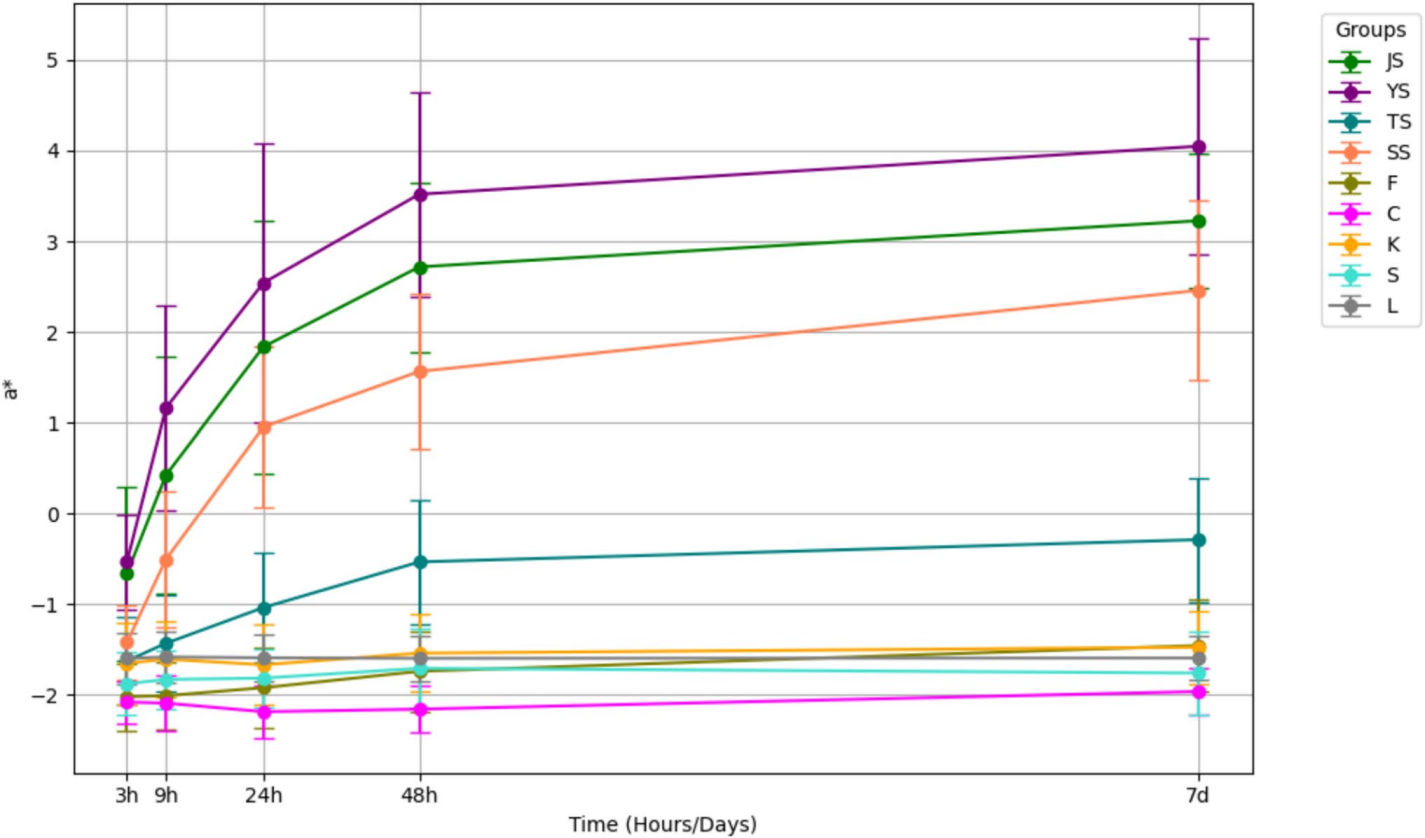
Change in a* values after consecutive immersion in nine different solutions

**Fig. 5:**
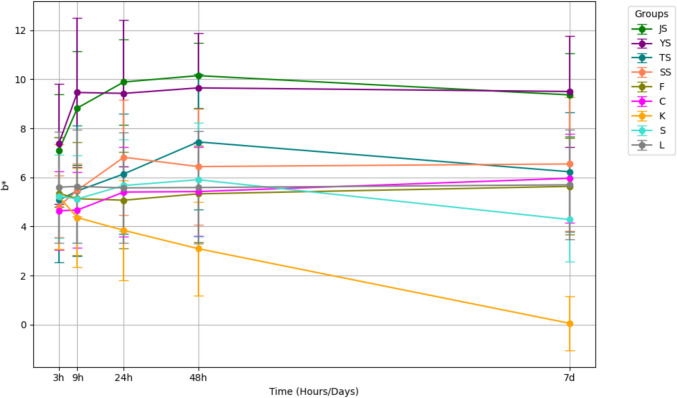
Change in b* values after consecutive immersion in nine different solutions

Colorimetric changes in L*, a*, and b* values revealed a decrease in lightness and an increase in redness in the Yangjo, Sashimi, and Jin groups (Figs. 3 and 4). Variations in yellowness/blueness were less prominent across all groups compared to changes in lightness and red/greenness (Fig. 5).

Absorbance and pH measurements also varied across groups, with Yangjo (absorbance, 1.648; pH, 4.80) and Sashimi (absorbance, 1.945; pH, 5.12) showing the highest absorbance values among the soy sauces (Table 3). Curry, Ketchup, and Sriracha exhibited the highest absorbance (3.000) but had more neutral or acidic pH levels (C: 6.13, K: 4.05, S: 4.21). The Fish sauce group had a low absorbance (0.175) and slightly acidic pH (5.81), while the Tsuyu soy sauce group displayed the lowest absorbance (0.031) and a pH of 5.35. The viscosity measurements indicated that, except for the Curry, Ketchup, and Sriracha groups, the viscosity of soy sauces and fish sauce was close to 1 mPa·s, which is comparable to the viscosity of water (Table 3).

Regarding the perceptibility and acceptability thresholds, the color differences revealed that all specimens from the Yangjo, Sashimi, and Jin soy sauce groups exceeded the perceptible threshold (ΔE_00_ = 0.8) within 3 h of immersion and surpassed the acceptable threshold (ΔE_00_ = 1.8) over the same period [15]. In the Tsuyu soy sauce group, most specimens exceeded the perceptibility threshold by 24 h, but fewer surpassed the acceptable threshold. The Curry group exhibited high resistance to staining, with only a few specimens exceeding the acceptable threshold after 7 days of immersion. In the Fish sauce group, all specimens surpassed the perceptible threshold within 3 h. However, in the Ketchup and Sriracha groups, all specimens exceeded the perceptible threshold within 7 days, but only a minimal proportion exceeded the acceptable threshold over the same period (Fig. 2).

**Table 3:** Absorbance, viscosity, and pH levels of the sauces used

Type	Absorbance	Viscosity (mPa·S)	pH
Jin soy sauce (JS)	1.533	1.6	5.05
Yangjo soy sauce (YS)	1.648	1.7	4.80
Sashimi soy sauce (SS)	1.945	2.4	5.12
Tsuyu soy sauce (TS)	0.031	1.3	5.35
Fish sauce (F)	0.175	2.2	5.81
Curry (C)	3.000	2866.3	6.13
Ketchup (K)	3.000	5558.5	4.05
Sriracha (S)	3.000	1716.7	4.21

## Discussion

This study demonstrated significant differences in the staining potential of various sauces, with distinct factors influencing the extent of tooth discoloration. The Yangjo, followed by the Sashimi and Jin soy sauces, exhibited the highest staining potential, which can be attributed to the dense concentrations of chromogens, particularly melanoidin, known to strongly bind to enamel surfaces and cause pronounced discoloration [6]. Melanoidins, also found in coffee, are produced during the Maillard reaction, which occurs during fermentation and heating, involving interactions between reducing sugars and free amino acids [16–18]. This reaction gives soy sauce its characteristic brown color and flavor and enhances its staining potential. Melanoidins contribute to the darker appearance of the tooth by binding to metal ions, such as calcium and proteins, on the enamel surface [19].

Previous studies have consistently highlighted the staining potential of soy sauces. Kang et al. [20] demonstrated that soy sauce caused more severe discoloration than other Korean fermented pastes, such as red pepper and soybean pastes, with the staining intensity increasing over time. Similarly, Chen et al. [21] reported a strong association between frequent soy sauce consumption and black tooth stains in preschool children. Their study revealed that children who regularly consumed soy sauce had 1.33 times more black-stained teeth than those who rarely or never consumed it. This finding underscores the critical role of dietary habits in extrinsic tooth discoloration and highlights soy sauce as a significant contributor to staining.

Acidity and pigment concentration emerged as key factors influencing the extent of discoloration. Soy sauces are mildly acidic, with pH levels conducive to enamel surface softening, which facilitates chromogen binding. The tooth enamel is primarily composed of hydroxyapatite crystals that are stable under neutral pH conditions. When the oral environment becomes acidic, these crystals begin to dissolve in a process known as demineralization due to the increased hydrogen ion concentration (H⁺); the hydroxyapatite lattice is disrupted in acidic conditions, leading to the release of calcium and phosphate ions from the enamel structure [22]. Previous studies have established that prolonged exposure to low pH or acidic solutions can enhance tooth discoloration by increasing enamel porosity, allowing pigments to adhere more effectively [13, 23].

The high absorbance values of soy sauces, indicative of their dense chromogen content, further reinforce their staining potential. The soy sauces demonstrated significantly higher absorbance levels than the fish sauce, which showed relatively lower staining potential in the current study. However, it is evident from the findings that acidity and absorbance alone do not fully explain the staining outcomes. Ketchup and sriracha exhibited lower pH and higher absorbance than soy sauces. However, they caused significantly less discoloration, indicating that other factors, such as the molecular composition of pigments, chemical interactions with enamel, and sauce viscosity, contribute to the complexity of tooth staining mechanisms.

The physical properties of the sauces, particularly their consistency and viscosity, may have played a role in the observed staining differences. Low-viscosity sauces, such as soy sauces and fish sauce, spread more uniformly over the tooth surface, allowing for greater contact and interaction with the enamel. This extended exposure time enhances the deposition and penetration of chromogens, leading to more pronounced discoloration. In contrast, thicker sauces like ketchup, sriracha, and curry exhibit reduced flow and less uniform surface coverage, which may limit their staining potential.

Among the soy sauces, Sashimi soy sauce, which has a slightly thicker consistency than other soy sauces, may better adhere to enamel surfaces, further increasing its staining potential. The prolonged contact time associated with higher viscosity can enhance the accumulation of pigments, promoting deeper and more intense discoloration. This raises important considerations regarding the extent of discoloration—whether it is limited to surface-level (extrinsic) staining or involves deeper penetration into the enamel matrix, potentially transitioning to intrinsic discoloration.

The staining potential of the sauces varied significantly depending on their composition, physical properties, and chemical characteristics. Among the soy sauces, Tsuyu soy sauce, which is typically diluted before consumption, exhibited the lowest absorbance due to its concentrated nature. Nonetheless, it caused noticeable staining over extended exposure periods, suggesting that sauces with low chromogen concentrations can lead to discoloration with sufficient contact time, thus highlighting the cumulative nature of chromogen interactions with the enamel.

In contrast, despite its vivid yellow color and high absorbance, curry showed remarkable resistance to staining, possibly due to its relatively neutral pH, which helped preserve enamel integrity by reducing erosion and preventing chromogen adherence. Curcumin, the primary pigment in curry, has been shown to stain materials such as polyoxymethylene brackets [24]. In addition, another study reported that orthodontic elastomeric chains made of polyurethane were more heavily stained by curry than coffee and wine, highlighting a strong interaction between curcumin and specific orthodontic dental materials [25]. This selective interaction may account for the minimal staining observed on the enamel in the current study. Similarly, with their vibrant red hues and low pH, ketchup and sriracha displayed surprisingly low staining potential, challenging the assumption that red-colored substances inherently cause tooth discoloration. The lack of a significant increase in redness compared to soy sauces suggests that staining is not solely dependent on color intensity but on the chemical behavior of chromogens and their interactions with enamel surfaces. Carotenoids, for example, are hydrophobic molecules that dissolve in lipids rather than binding strongly to hydrophilic surfaces, such as the enamel [26, 27]. Curcumin also binds to the tooth surface, but the inherent brown color of melanoidin likely plays a key role in darkening the tooth surface [28]. It should be noted that the absence of statistically significant staining in some groups may have been influenced by limited sample size, potentially masking smaller effects that the study was underpowered to detect.The findings of this study have important clinical implications, particularly for dietary recommendations aimed at minimizing tooth discoloration. While soy sauces, especially Yangjo, Sashimi, and Jin, pose a significant risk for tooth staining, sauces like curry, ketchup, and sriracha may offer alternative options for individuals concerned with esthetic dental outcomes. For patients undergoing tooth whitening or other esthetic dental treatments, avoiding highly pigmented and acidic sauces, such as soy sauces, may help maintain treatment results and prevent rapid discoloration.

In addition, the results of this study suggest that viscosity and contact time should be considered when assessing the staining potential of foods and beverages. Understanding the interplay between the physical properties, chemical composition, and enamel interactions can aid in developing strategies to manage and mitigate extrinsic discoloration. These insights are particularly relevant for the formulation of preventive care protocols and dietary guidelines tailored to individual esthetic needs.

While this study provides valuable insights into the staining potential of various sauces, certain aspects warrant further exploration. The experiments were conducted under in vitro conditions, which may not fully capture the complexity of the oral environment, including factors such as saliva flow, biofilm formation, and individual oral hygiene habits. Saliva plays a crucial role in modulating tooth discoloration through its natural buffering capacity, while the salivary pellicle serves as a protective barrier, preventing enamel demineralization and minimizing pigment deposition on the tooth surface [29]. Salivary proteins, conversely, may promote staining, as previous studies have shown that the interaction between salivary components and dietary substances can enhance discoloration [30, 31]. Therefore, the presence of saliva may substantially impact the discoloration outcomes.

Polished bovine enamel differs from natural human enamel because it lacks a surface texture and an intact pellicle layer, which may affect stain adhesion. In addition, the sauces were tested in their undiluted forms, whereas in real-world settings, they are typically diluted or consumed with other foods. Dietary habits, such as consumption frequency, and the balance between staining and tooth brushing, were also not considered. Lastly, the sample size was determined based on practical feasibility rather than strictly adhering to calculated value. While meaningful differences were still observed between several groups, this limitation should be considered when interpreting comparisons that did not reach statistical significance. Future research should address these factors for a more comprehensive understanding of the tooth discoloration mechanisms.

## Conclusions

This study demonstrated significant differences in the staining potential of various sauces, with Yangjo soy sauce exhibiting the highest discoloration effects on bovine enamel specimens. The pronounced staining capacity of soy sauces is likely due to their dense chromogen content, particularly melanoidin, formed during the Maillard reaction. In contrast, despite its vivid yellow color, curry caused minimal staining, possibly due to the weak interaction between curcumin and enamel. Similarly, ketchup and sriracha, though rich in bright red pigments, resulted in relatively low levels of discoloration. These findings suggest that the chemical nature of pigments and their affinity for enamel are more critical determinants of staining than color intensity alone. Moreover, the results highlight the complex interplay of pigment concentration, acidity, viscosity, and exposure duration in tooth discoloration, emphasizing the importance of dietary counseling, particularly for patients undergoing esthetic dental treatments such as whitening. It should also be acknowledged that extrinsic staining from pigmented foods is not necessarily cumulative, and that the in vitro immersion model may not fully reflect the intermittent and dynamic nature of staining in the oral environment. Further investigation is needed to better simulate clinical conditions and assess the long-term staining effects of dietary pigments on enamel.

## Data Availability

The data that support the findings of this study are available under reasonable request.

## References

[1] Barner A, Frank C, Shipton L. Tooth discoloration in a patient with multidrug-resistant tuberculosis. Clin Infect Dis. 2016;62:664–5. 10.1093/cid/civ997 26908847

[2] Plotino G, Buono L, Grande NM, Pameijer CH, Somma F. Nonvital tooth bleaching: a review of the literature and clinical procedures. J Endod. 2008;34:394–407. 18358884 10.1016/j.joen.2007.12.020

[3] Arıkan V, Sarı S, Sonmez H. Bleaching a devital primary tooth using sodium perborate with walking bleach technique: a case report. Oral Surg, Oral Med, Oral Pathol, Oral Radiol Endodontol. 2009;107:e80–4. 10.1016/j.tripleo.2009.01.050 19426913

[4] Kim S, Larnani S, Taymour N, et al. Effect of coffee roasting level on tooth discoloration. J Oral Sci. 2024. 10.2334/josnusd.24-0287. 39647855 10.2334/josnusd.24-0287

[5] Kim S, Song JS, Yoon J, Garcia-Godoy F, Park YS. Influence of coffee characteristics on tooth discoloration. Am J Dent. 2024;37:171–6. 39186595

[6] Kim S, Son JE, Larnani S, et al. Effects of tea and coffee on tooth discoloration. Ital J Food Sci. 2024;36:64.

[7] Yang S, Fan W, Xu Y. Melanoidins present in traditional fermented foods and beverages. Compr Rev Food Sci Food Saf. 2022;21:4164–88. 36018462 10.1111/1541-4337.13022

[8] Lee YS, Homma S, Aida K. Characterization of melanoidin in soy sauce and fish sauce by electrofocusing and high performance gel permeation chromatography. Nippon Shokuhin Kogyo Gakkaishi. 1987;34:313–9.

[9] Mert B. Using high pressure microfluidization to improve physical properties and lycopene content of ketchup type products. J Food Eng. 2012;109:579–87.

[10] Kim S, Ha TY, Hwang IK. Analysis, bioavailability, and potential healthy effects of capsanthin, natural red pigment from Capsicum spp. Food Rev Intl. 2009;25:198–213.

[11] Prasad S, Tyagi AK, Aggarwal BB. Recent developments in delivery, bioavailability, absorption and metabolism of curcumin: the golden pigment from golden spice. Cancer Res Treat. 2014;46:2–18. 24520218 10.4143/crt.2014.46.1.2 PMC3918523

[12] Kim S, Lee C-H, Ma S, Park Y-S. Whitening efficacy of toothpastes on coffee-stained teeth: an enamel surface analysis. Int Dental J. 2024;74(6):1233–8. 10.1016/j.identj.2024.02.006 PMC11551553 38614882

[13] Kim S, Chung SH, Kim RJY, Park Y-S. Investigating the role of chlorogenic acids and coffee type in coffee-induced teeth discoloration. Acta Odontol Scand. 2023;82:1–8. 37565724 10.1080/00016357.2023.2245880

[14] Tanaka K, Someya T, Kawada E, et al. In vitro wear behavior of restorative resin composites against bovine enamel. Dent Mater J. 2020;39:915–23. 31694995 10.4012/dmj.2018-297

[15] Paravina RD, Ghinea R, Herrera LJ, et al. Color difference thresholds in dentistry. J Esthet Restor Dent. 2015;27:S1–9. 25886208 10.1111/jerd.12149

[16] Det-Udom R, Gilbert C, Liu L, et al. Towards semi-synthetic microbial communities: enhancing soy sauce fermentation properties in B. subtilis co-cultures. Microb Cell Fact. 2019;18:101. 31159886 10.1186/s12934-019-1149-2 PMC6547557

[17] Miwa M, Watanabe T, Kawasumi T, Hayase F. Protective effects of melanoidins derived from soy sauce and soy paste on NO-induced DNA damage. Food Sci Technol Res. 2002;8:231–4.

[18] Wang H, Jenner AM, Lee C-YJ, et al. The identification of antioxidants in dark soy sauce. Free Radical Res. 2007;41:479–88. 17454130 10.1080/10715760601110871

[19] Shaheen S, Shorbagi M, Lorenzo JM, Farag MA. Dissecting dietary melanoidins: formation mechanisms, gut interactions and functional properties. Crit Rev Food Sci Nutr. 2022;62:8954–71. 34137312 10.1080/10408398.2021.1937509

[20] Kang H-K, Lim H-J. The relation between korean traditional fermented food and discoloration on bleached tooth. J the Korea Acad-Ind Coop Soc. 2012. 10.5762/KAIS.2012.13.10.4717.

[21] Chen X, Zhan J-Y, Lu H-X, et al. Factors associated with black tooth stain in Chinese preschool children. Clin Oral Invest. 2014;18:2059–66. 10.1007/s00784-013-1184-z 24430339

[22] Dawes C. What is the critical pH and why does a tooth dissolve in acid? J Can Dent Assoc. 2003;69:722–4. 14653937

[23] Hutami S, Triaminingsih S, Indrani D. Effect of tooth immersion in the coffee drink with different types of coffee roast temperature on tooth discoloration. J Phys: Conf Ser. 2018;1073: 032026.

[24] Wriedt S, Schepke U, Wehrbein H. The discoloring effects of food on the color stability of esthetic brackets–an in-vitro study. J Orofac Orthop. 2007;68:308–20. 17639279 10.1007/s00056-007-0640-2

[25] Chung H-J, Lim S-A, Lim H-K, Jung S-K. Perceptual and quantitative analysis of discoloration of orthodontic elastomeric chains by food. BMC Oral Health. 2023;23:124. 36829133 10.1186/s12903-023-02825-2 PMC9951536

[26] Háda M, Nagy V, Deli J, Agócs A. Hydrophilic carotenoids: recent progress. Molecules. 2012;17:5003–12. 22547321 10.3390/molecules17055003 PMC6268248

[27] Gutiérrez-Camacho J, Galavíz L, Sanchez-Balderas G, et al. In-vitro silanization of dental enamel to prevent demineralization. Odovtos - Int J Dental Sci. 2021;24:353–63.

[28] Inchingolo F, Inchingolo AD, Latini G, et al. The role of curcumin in oral health and diseases: a systematic review. Antioxidants. 2024;13:660. 38929099 10.3390/antiox13060660 PMC11200638

[29] Chawhuaveang DD, Yu OY, Yin IX, et al. Acquired salivary pellicle and oral diseases: a literature review. J Dent Sci. 2021;16:523–9. 33384841 10.1016/j.jds.2020.10.007 PMC7770358

[30] Carpenter GH, Pramanik R, Proctor GB. An in vitro model of chlorhexidine-induced tooth staining. J Periodontal Res. 2005;40:225–30. 15853968 10.1111/j.1600-0765.2005.00791.x

[31] Proctor GB, Pramanik R, Carpenter GH, Rees GD. Salivary proteins interact with dietary constituents to modulate tooth staining. J Dent Res. 2005;84:73–8. 15615880 10.1177/154405910508400113

